# Response to Flavored Cartridge/Pod-Based Product Ban among Adult JUUL Users: “You Get Nicotine However You Can Get It”

**DOI:** 10.3390/ijerph18010207

**Published:** 2020-12-30

**Authors:** Jessica M. Yingst, Candace R. Bordner, Andréa L. Hobkirk, Brianna Hoglen, Sophia I. Allen, Nicolle M. Krebs, Kenneth R. Houser, Craig Livelsberger, Jonathan Foulds

**Affiliations:** 1 Department of Public Health Sciences, Penn State University College of Medicine, Hershey, PA 17033, USA; jyingst@phs.psu.edu (J.M.Y.); sallen3@phs.psu.edu (S.I.A.); nkrebs@pennstatehealth.psu.edu (N.M.K.); clivelsberger@phs.psu.edu (C.L.); jfoulds@psu.edu (J.F.); 2 Department of Psychiatry and Behavioral Health, Penn State University College of Medicine, Hershey, PA 17033, USA; ahobkirk@pennstatehealth.psu.edu (A.L.H.); bhoglen@pennstatehealth.psu.edu (B.H.); khouser@pennstatehealth.psu.edu (K.R.H.)

**Keywords:** electronic cigarette, perceptions, mixed methods research, flavored tobacco products, food and drug administration, disposable e-cigarettes, tobacco control policy

## Abstract

In order to curb increasing youth electronic cigarette (e-cig) use, the United States Food and Drug Administration (FDA) banned the sale of flavored cartridge/pod-based products in February 2020. This mixed-methods study aimed to evaluate the impact of the FDA ban on adult JUUL users. The samples of current adult JUUL users were surveyed via Amazon Mechanical Turk at three time-points *n* = 76 (Sample 1); *n* = 128 (Sample 2); *n* = 86 (Sample 3) before and after the FDA flavored/pod ban. The participants were asked to report the JUUL flavored pod used most often and answer questions on purchasing generic pods or refilling (Quantitative). JUUL users were then interviewed in order to explore their perceptions and behaviors related to the FDA ban of flavored cartridge/pod-based products (Qualitative; *n* = 16). Quantitative data analysis evaluated the differences in variables by time-point. Qualitative data were coded into themes while using the constant comparative method. We found a significant decrease in the use of mint pods (43.4%, 22.7%, 16.3%) (*p* < 0.01), while there was a significant increase in the use of menthol pods (6.6%, 26.6%, 37.2%) (*p* < 0.01). Themes that emerged from the qualitative data included switching from mint to menthol pods, refilling pods, and switching to other products that are available in the desired flavors, such as generic pods or disposable e-cigs. Future research is needed in order to evaluate the impact of these behaviors on public health.

## 1. Introduction

Electronic cigarettes (e-cigs) are a diverse class of tobacco products that aerosolize a nicotine-containing liquid that is directly inhaled by the user. E-cig devices are available in many variations and they have rapidly evolved over time. The first e-cig devices were simple, small devices shaped like a combustible cigarette (cigalikes) that housed the nicotine-containing liquid in cartridges. Advances in technology lead to more complex, larger pen and box-shaped devices that utilized refillable tanks, or reservoirs, to house the nicotine liquid (advanced/mods) [1]. The most recent evolution of e-cigs, which are referred to as pod-mods, are small, discrete devices that utilize interchangeable pods to house the nicotine-containing liquid, typically in a salt form, which is less harsh [2]. These devices have a sleek design, user-friendly functionality, and an array of flavor options [3]. They are also known to deliver nicotine in doses that are higher than many earlier devices [4,5,6]. This type of device was made popular by the market leader called JUUL [7].

In 2018, 14.9% of adults in the United States reported ever using an e-cig, and 3.2% reported current use [8]. Many adults report initiating e-cig use as a way to quit cigarette smoking [9,10]; however, the impact of e-cig use on quitting smoking has been controversial in the literature [11,12,13]. Some studies have reported that e-cig use is not associated with increased success in quitting [14,15], while other studies have found that e-cigs can be effective aids in quitting [16,17,18]. A recent, large randomized controlled trial that was conducted in the United Kingdom found that those using e-cigs were almost twice as likely to remain abstinent from cigarettes at one year, when compared with those using nicotine replacement therapy [17]. Of importance, studies have reported that flavored products are used by the majority of adult users [19,20,21], and could be associated with a successful transition from cigarettes to e-cigs [22,23,24,25].

While the use of e-cigs may help adult smokers to reduce or quit smoking, the wide availability of e-cigs poses a risk to youth. In 2020, 19.6% of high school students and 4.7% of middle school students in the United States reported using an e-cig in the past 30 days, which makes e-cigs the most widely used tobacco product in this age group [26]. Most youth e-cig users report using pod-based products [26,27], like JUUL, which are capable of delivering nicotine in high doses that are similar to a cigarette [5,6]. In addition, the majority of youth e-cig users reported initiating e-cig use because of the appealing flavor options [28]. One study reported that 77.9% of adolescent and 90.3% of young adult users in the United States reported appealing flavors as a reason for e-cig use [28]. Together, these findings suggest that flavored products available with devices that are capable of delivering large amounts of nicotine may be driving youth e-cig use.

In an effort to curb youth uptake, several steps were taken to reduce youth access to flavored products in the United States. First, JUUL Labs Inc. voluntarily removed mango, crème, fruit, and cucumber flavored products from retail stores in November 2018 and later online in October 2019. Soon after, in November 2019, JUUL also completely removed mint flavored products from the market [29]. These steps were taken in response to criticism that JUUL received for their marketing practices, which were seen to promote youth use through the use of young models, brightly colored designs, multitude of flavored products, and use of the social media platform for promotions [7]. Most recently (February 2020), the United States Food and Drug Administration (FDA) banned the sale of all flavored cartridge/pod-based products. This ban included “any small, enclosed unit (sealed or unsealed) designed to fit within or operate as part of an ENDS product”. Of importance, the ban did not include tobacco or menthol flavored pod-based products or flavored liquid products that were used in tank-based devices or flavored disposable products [27]. Although similar, mint was distinguished from menthol, because it is not a traditional cigarette flavor and it was popular among youth [30].

Because flavored cartridge/pod-based products that are widely-used by both adults and youth are no longer available on the market, it is important to evaluate the impact on users. This explanatory sequential mixed-methods study aimed to evaluate the impact of the flavored cartridge/pod-based ban among adult JUUL users specifically. First, the samples of current adult JUUL users were surveyed at varying time points (before and after the FDA flavor ban) in order to measure changes in the proportion of users reporting the use of flavored pods. In addition, this study also aimed to explore use behaviors that could result from the changes in flavored pod availability. In order to further explain the quantitative results, qualitative interviews were conducted with adult JUUL users to explore their perceptions of the flavored cartridge/pod-based ban and understand how it affected their use of these products.

## 2. Methods

**Figure 1:**
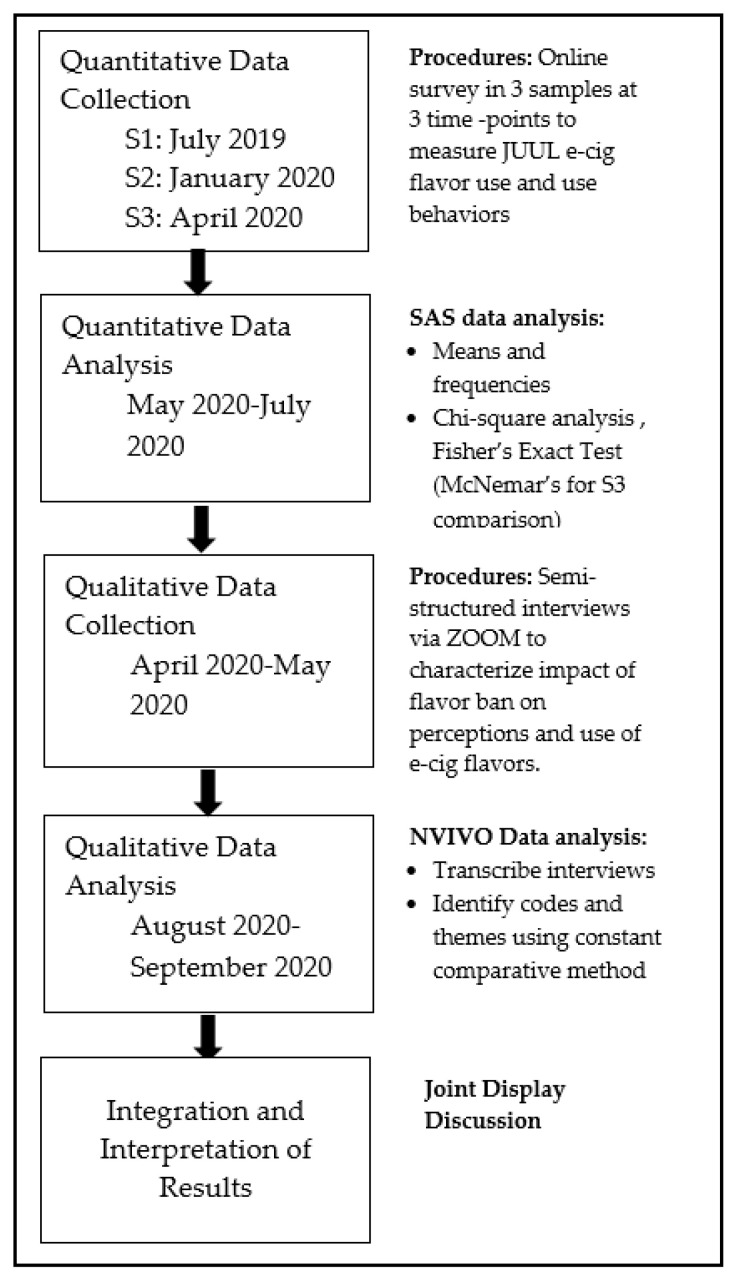
Procedural Diagram.

This explanatory sequential mixed-methods study of current JUUL users included a quantitative online survey at three time points and qualitative interviews. The results from the quantitative and qualitative data were separately analyzed and the results were integrated for interpretation. Figure 1 depicts a procedural diagram for the study.

### 2.1. Quantitative Data Collection

#### 2.1.1. Participants

Cross-sectional samples of current JUUL users (aged ≥ 18 years and reporting JUUL use in the past 30 days) completed online surveys regarding their JUUL use. Survey data were collected at three time points. The first sample of participants (Sample 1) was surveyed prior to JUUL flavored pod restrictions, when flavored pods were only available online (July 2019). The second sample of participants (Sample 2) was surveyed in January 2020, after the removal of JUUL flavored pods, including mint, from the market. The third sample of participants (Sample 3) was surveyed in April 2020, after the FDA enacted the ban of all flavored cartridge/pod-based products.

All participants were recruited via Amazon Mechanical Turk (MTurk), an online labor market that uses crowdsourcing in order to recruit diverse adult (18 years of age of older) samples for online tasks that require human intelligence. MTurk is an efficient and convenient method for recruiting smokers and e-cigarette users for survey research, including JUUL users [31,32], and studies have shown that the data that were collected via MTurk for social science research are valid and internally consistent, with good test-retest reliability [33,34,35]. In order to improve the reliability and validity, the respondents were restricted to those in the United States with job approval ratings on MTurk of at least 98%. All of responses were briefly reviewed by the researchers before approval and payment was provided to the user. Duplicate participants at each time-point were identified by the MTurk Worker ID and the duplicate record was removed. In order to verify that participants were actual users of JUUL, responses to open-ended questions and time of use questions were examined during data cleaning. In addition, the users were asked to upload a photograph of their actual JUUL device. The participants who provided data that appeared falsified (i.e., repeated number use, logically impossible responses) and/or who did not provide a picture of their device were excluded from the data.

All of the participants gave their informed consent for inclusion before they participated in the study. The study was conducted in accordance with the Declaration of Helsinki, and the protocol was approved by the Penn State College of Medicine Institutional Review Board (IRB #12315).

#### 2.1.2. Measures

The participants were asked to report demographic characteristics, including age and gender. They were also asked questions regarding their smoking history, including whether they have ever smoked cigarettes and the number of days/months/years since they last smoked cigarettes. Those reporting cigarette smoking in the past 30 days were considered to be current smokers.

Questions relating to JUUL use included the number of days used in the past 30 days and the number of times used per day (“time” defined as use lasting either 10 min. or 15 puffs). In addition, questions were included to measure the use of flavored pods and the location of their purchase (in stores vs. online). The participants were asked, “During the past 30 days, which flavor JUUL refill pod have you used most frequently?” and “Where do you purchase your JUUL pods most often?” Finally, the participants were asked whether they ever refill their single-use JUUL pods with nicotine liquid and if they purchase generic pods, defined as pods which are compatible with the JUUL device, but not manufactured by JUUL. Those who purchased generic pods were asked to select the reason they chose to purchase generic pods from a list of options (less expensive, additional flavor options, easier to purchase, are refillable, hold more liquid, available in 0% nicotine, and other).

For the last sample of survey participants (S3), we asked the participants to retrospectively report the JUUL flavored pod that they used most often prior to JUUL removing flavors from the market, in addition to the flavor of the JUUL pod that they used at the time of survey completion. The participants were asked, “Prior to JUUL stopping the sales of flavored pods, which JUUL flavor did you use most often?”.

#### 2.1.3. Data Analysis

The participants who reported their basic demographic information and their preferred flavor were included in the analysis. Means and frequencies were used in order to describe the participants and their use characteristics. Chi-square analysis and one-way ANOVA were used to determine the differences in variables of interest by survey wave (between-subject). Fisher’s Exact Test, with the Freeman–Halton extension [36], was used in order to evaluate differences in proportions when the expected frequency in any cell was less than 5 [37]. McNemar’s test was used to look at the within-subject changes in flavor for the S3 retrospective and current flavored pod usage.

### 2.2. Qualitative Data Collection

#### 2.2.1. Participants

The participants were adult (age 21+) JUUL users who reported JUUL use in the past 90 days. An inclusion period of 90 days was selected to ensure that the sample was not biased towards participants who continued JUUL use after the FDA ban. The participants were recruited from a database of potential research participants, maintained by the Penn State Center for Research on Tobacco and Health, who previously indicated JUUL use and indicated interest in participating in future research studies. This database included participants from the quantitative study discussed above. Those who indicated interest in future research were sent an email describing the study and instructing them to call the research center if they were interested in participating. Those that were interested were screened and those eligible were sent an email with their scheduled interview time, a summary explanation of the research, and a link to join the researcher via ZOOM for Healthcare, a HIPAA compliant online videoconferencing service, at the scheduled visit time.

All of the participants gave their informed consent for inclusion before they participated in the study. The study was conducted in accordance with the Declaration of Helsinki, and the Penn State College of Medicine Institutional Review Board approved the protocol (IRB #14507).

#### 2.2.2. Measures

The researcher started the interview by asking the participant to report on basic demographic characteristics and to show their JUUL device in order to confirm use. The researcher then used the interview guide to ask the participant a series of questions regarding their JUUL use, including how they were impacted by the ban on flavored cartridge/pod-based based products. All of the interview visits were audio and video recorded while using ZOOM for Healthcare.

#### 2.2.3. Data Analysis

The means and frequencies were used to describe the demographic data. First, all of the interviews were transcribed verbatim into transcripts, and then imported to NVIVO 10 software (QSR International, Burlington, MA, USA) for coding and analysis. Researchers then generated a codebook containing 10 a priori themes that were based on the literature. Transcripts were then reviewed while using the constant comparative method [38] and the codebook was refined through comparison and discussion. Next, each researcher coded the same four transcripts (25% of the data) while using the finalized codebook and inter-rater reliability (IRR) was calculated between the researchers (J.M.Y and C.R.B.) with a reliability standard of k > 0.8 in order to ensure consistency in coding. All of the data presented in this study were coded into the theme, FDA flavor ban. Sub-themes for the FDA flavor ban theme included no impact, switching to other available pods, switching to other available products, and continuing use of flavored pod-based products illicitly.

#### 2.2.4. Data Integration

Quantitative and qualitative data were integrated using a joint display. A joint display integrates the quantitative and qualitative data in a table, allowing further insight into the data that could not be understood when separately analyzing the data [39].

## 3. Results

### 3.1. Quantitative Study

Two hundred and ninety participants met the study inclusion criteria for the quantitative study (*n* = 76 (S1); *n* = 128 (S2); *n* = 86 (S3)). The participant characteristics can be found in Supplemental Table S1. There were no significant differences for these demographic variables between the survey time-points.

**Figure 2:**
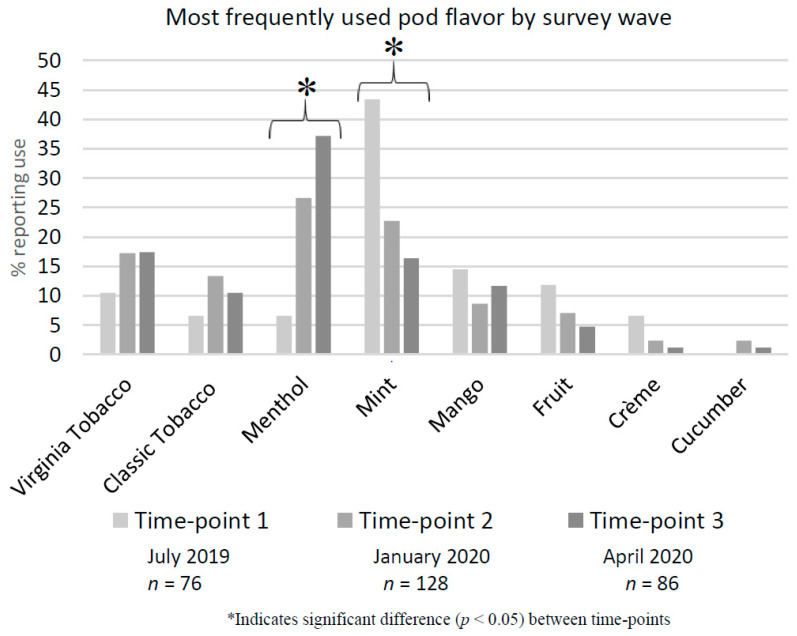
Most frequently used pod flavor by survey time-point.

At survey time-point 1 (S1 July 2019), mint flavored pods were the most commonly used (43.4%). There was a significant decrease in the use of mint pods from S1 to S3 (April 2020) (43.4%, 22.7%, 16.3%) (*p* < 0.01) (Figure 2). In addition, there was a significant increase in the use of menthol pods from S1 to S3 (6.6%, 26.6%, 37.2%) (*p* < 0.01), which makes menthol the most commonly used flavor by S3. While the use of other flavored pods slightly decreased over time, these differences were not significant. Of interest, there was not a significant decrease in use of the popular flavor mango that should not have been legally available at S3 (14.5%, 8.6%, 11.6%, *p* = 0.42). Similar trends were found in a subsample of young adult users (18–25 years) (*n* = 63). Mint use decreased (52.9%, 30.4%, 21.7%) (*p* = 0.13), however, not significantly, while menthol use increased (5.9%, 26.1%, 43.5%) (*p* = 0.03).

At S3 only, the participants were asked to report their current flavored pod used and the flavored pod used most often prior to the FDA ban. With this within-subject analysis, there were significant decreases in the use of mint, mango, and fruit pods, and significant increases in the use of Virginia tobacco, classic tobacco, and menthol pods (Table 1). There were no changes in the use of crème and cucumber pods.

In addition to changes in flavored pod usage, this study also measured the impacts on use behaviors. There was a significant difference over time in the proportion of participants who reported refilling their pods (19.7%, 34.4%, 11.6%) (*p* < 0.01) (Table 2). There were no differences in the proportion of users who reported using generic pods (*p* = 0.15) or their reason for purchasing generic pods (*p* = 0.29). In addition, there was not a difference in the proportion of users who bought their pods online; however, at S2, when flavored pods were only available online, almost half of the participants (46.1%, *n*= 66) reported purchasing pods online.

### 3.2. Qualitative Results

The characteristics of the sample (*n* = 16) can be found in Supplemental Table S1.

Many of the participants reported that the recent ban of flavored pods did not affect their use. One participant said, “*It hasn’t really affected me because I just smoke the tobacco flavor pod*” (female, age 24, 2 CPD). Others noted that they were not affected because they do not prefer the fruit flavors. One said, *“I’ve always only preferred the menthol and that’s the only one that I’ve used. I mean I’ve tried [flavors] in the beginning cause it came with them, but never been a fan of the fruity flavors”* (male, age 33, 20 CPD). Another participant said, “*I know some people really get hung up on those flavors. It’s never been a huge draw for me*” (male, age 43, 1 CPD). Others were not affected, because they continued use of the banned flavors. These users reported stockpiling their pods, saying “*so it made me kind of hoard them*” (male, age 38, former smoker). Others reported continuing to buy the banned products from stores illegally selling them. One participant said, “*there were like a few stores I had around here that were like sketchy and like secretly had them*” (female, age 26, former smoker).

Other participants noted that, while they were affected by the ban of flavored pods, they were able to quickly adapt to the available pods, mainly because the products still contained nicotine. One said, “*I think the mint flavor was like my favorite one… and then when they banned that, um, I don’t know, you just adapt. Like we all hated the other flavors that they left us, but like we’ve adapted... Like, at first I didn’t like the menthol flavor at all. And now I prefer it because, well, [it’s] one of two flavors that are around. So you get nicotine however you can get it*” (female, age 29, 2 CPD). Other participants said, “*I did really like the mint ones. That was my go to, but the menthols are just as good*” (female, age 24, former smoker) and, “*I mean, once you start, you try the JUUL and you’re like, okay mango’s good. This is amazing… but the point is you’re addicted to nicotine. It doesn’t matter at that point. I’m just like oh… I don’t have my crème B. It’s just like you tell an alcoholic, you know, sorry we don’t have your [drink]. We only got the peach or something. It’s like I don’t care. Give me the stuff with alcohol or nicotine*” (male, age 34, never smoker). Finally, several participants noted how the ban of flavored pods has lead them to use other flavored products, including disposable devices. One participant said, *“They still sell all sorts of flavors and disposable devices, which is even easier to access than buying a JUUL kit*” (female, age 59, never smoker). Another participant noted that they use the available JUUL pod, but treat themselves with flavored disposable products. They said, “*When I feel like I want to reward myself, I tell myself that I’ll get like a flavored Puff Bar, something like that. But they’re a little bit more expensive*” (male, age 23, 3 CPD). Another participant reported the use of generic flavored pods. They said, “*It has completely changed my buying habits. I always a hundred percent of the time, if it’s available, choose a generic that has a better flavor over the actual JUUL pod*” (female, age 28, 10 CPD). These participants expressed confusion over differences in which flavored products were allowed to be sold. These participants said, “*I’m kind of confused on what happened with that situation. Like, how come other vape companies can use like different flavors but JUUL can’t*” (male, age 23, 3 CPD) and “*So that just makes no sense to me. I go and they’ve told JUUL, you can’t sell your customers flavors, but I can buy flavors in all these other brands that are disposable. So that’s just kind of punitive if you ask me*” (female, age 59, never smoker).

Table 3 displays the merged results for the quantitative and the qualitative findings.

## 4. Discussion

Among adult JUUL users, we found that the use of flavored JUUL pods was significantly impacted by the removal of flavored JUUL pods from the market, and the later FDA ban on the sales of all brands of flavored cartridge/pod-based products. Prior to JUUL removing their flavored products from the market, mint was the most popular flavored pod, being used by more than 43% of users. Following the FDA ban, the data from the three time-points showed a significant decrease in the use of mint pods and a significant increase in the use of menthol pods, which makes it the most popular flavored pod (34.8%). These findings were consistent with the within-subject data that were collected from participants at time-point 3. In addition, the findings from our small study were also supported by retail market data, which showed a dramatic increase in menthol flavored cartridge/pod-based product sales following JUUL’s removal of mint flavored pods from the market [29,40]. One study reported that, by May 2020, menthol dominated the market, with 61.8% of prefilled cartridge/pod-based sales [29]. Many participants perceived that the shift from mint, a flavor removed from the market, to menthol, an available flavor, was easy, because both products contained nicotine, the real driver behind their continued use. This is an important finding, because it suggests the willingness of users to switch to the flavors that are available [40].

While the use of some flavors significantly changed after the FDA ban, we found that other flavors, including some that were banned, did not change. This same pattern was also found in a study that evaluated flavor use after the ban of flavored products in San Francisco [41]. This suggests that users are still able to access banned flavored pods in some way, especially since the retail data suggest that users are not purchasing these products in stores [29]. First, it is possible that users stockpiled JUUL pods in anticipation of the ban, as indicated in the qualitative data, and they were still using pods from their own supply at the time of the survey. Additionally, it is possible that users were able to purchase JUUL pods from stores who continued to sell them illegally or obtain pods from other countries that are able to legally sell JUUL flavored pods. In addition, it is also possible that users could have purchased counterfeit pods that are marketed as JUUL [42]. Finally, it is possible that users misinterpreted the question and instead of reporting use of JUUL brand pods, they were reporting the flavor of generic or refilled JUUL-compatible pods. It will be important for future studies to evaluate how users are able to access the banned flavors.

We found a slight increase in the number of users reporting generic pods use from S1 to S3, with approximately 12% of users reporting the use of generic brand flavored cartridge/pod-based products after the FDA ban. This upward trend in generic pod use could be due to a lack of compliance with the FDA regulations by the companies that produce these generic products. While companies not in compliance risk enforcement action by the FDA [27], enforcement could be delayed due to the onset of the COVID-19 pandemic, leaving these products to remain on the market. This shift from brand name products, like JUUL, with known quality control methods to generic products is concerning. Recently, there was an outbreak of a serious lung illness, called EVALI (e-cigarette or vaping product use-associated lung injury), which was later identified as being caused by black market THC cartridges that contained Vitamin E acetate [43,44]. While EVALI was not linked with the use of nicotine e-cig pods, it is possible that a similar outbreak could occur among nicotine e-cig users if the use of generic products or illicit products continues outside of quality controls that are required for FDA Premarket Tobacco Product Application (or PMTA) approval.

In addition, we found a significant difference over time in the proportion of users who reported refilling their single-use JUUL pods, a behavior that is warned against for safety and quality reasons [45]. This involves purchasing an e-cig liquid that is not intended for use in JUUL products and adding that liquid to a JUUL pod. JUUL pods are designed for one-time use, so this is not easy, but consumers can find instructional videos and posts online. Our data showed that, when flavored JUUL pods were no longer on the market, there was an increase in refilling. Subsequently, with the FDA ban, there was a sharp decrease in reported refilling. This decrease in refilling could be attributed to participants learning to find other sources of flavored products, such as purchasing generic brand flavored pods, reducing the need to refill their JUUL pods with the flavors that they like. It is also possible that users switched to other flavored product types. While flavored cartridge/pod-based products were banned by the FDA, flavored disposable e-cig products were not. A recent study examining retail data showed an increase in the use of mint flavored disposable products from 2017 to 2020 [29], and the National Youth Tobacco Survey reported a 1000% increase in the use of disposable products from 2019 to 2020 [26]. In addition, a study of internet searches reported a sharp increase of searches pertaining to disposable products, like Puff Bar [26,46]. Switching to products that are available with flavors was discussed by participants during the qualitative interviews, and many perceived the differences in what flavored products could and could not be sold to be confusing. This loophole in regulation is particularly concerning for youth populations who may still have access to flavored products.

The current study was not able to assess whether the FDA ban on flavored products led adult users to return to cigarette smoking. Future longitudinal studies are needed in order to further evaluate the impact of flavored product bans on returning to smoking. Other limitations include the use of a small convenience sample for the quantitative portion of the study. We conducted post-hoc power analysis to address this limitation, and determined that the study had sufficient power to detect small to medium effects (phi ≥ 0.2), while it was under powered for detecting small effects sizes (phi < 0.2). Given the effect sizes that were found for our chi-square analyses assessing change in flavor proportion across the three time-points, we had sufficient power to detect small to medium effects in comparisons of mint, menthol, and mango (phi ≥ 0.2, estimated power ≥ 87%) and the study was underpowered to detect small effects in classic tobacco, Virginia tobacco, fruit, crème, and cucumber (phi < 0.2, estimated power < 45%). Future research with larger sample sizes will help to elucidate the more nuanced effects that regulations and market changes might have on flavor use over time.

Additionally, the study was limited by the recruitment of the sample while using an online platform, called MTurk. The MTurk participants are not representative of the general population; therefore, we are not able to make any population-based inferences that are based on these results [47]. However, MTurk is an ideal platform to recruit JUUL users, since those completing tasks tend to be younger, with an over-representation of substance users [33,48]. In addition, this study focused on adult JUUL users. This was due to the age restrictions of the MTurk platform, which only allows for those 18 and older to have an account. Because the youth population is of interest, we completed a sub-subsample analysis to compare the impacts on young adults to the entire sample. These results were similar, which suggested that young adult and adult users respond similarly to flavor restrictions. Future studies are needed to evaluate the impact of these restrictions among a youth and young adult population.

Finally, the data that were collected for the quantitative part of the study was cross-sectional, collected from unique samples at three time points. The data collected were not collected longitudinally from the same participants over time, which would have yielded richer data and could have provided insight on whether users continued JUUL use or returned to smoking after the FDA ban. Finally, while some of the participants completed both the quantitative and qualitative parts of the study (*n* = 7, 43.8% of the qualitative interviews completed), the majority of qualitative interviews were completed by participants who were not included in the quantitative part of the study. However, the samples that were recruited for both parts of the study were very similar in regards to demographics and JUUL use characteristics, likely minimizing any issues.

## 5. Conclusions

Availability restrictions, such as the removal from shelves or outright bans, of specific e-cig liquid flavored cartridge/pod-based products led to only partial reductions in the use of banned flavors. Where loopholes or a lack of enforcement exists, some manufacturers seek to exploit these loopholes, and users adjust their behavior in order to acquire either banned flavors or the closest available e-cig liquid (e.g., refilling pods with flavored liquids no longer sold by JUUL or switching from mint to menthol flavored JUUL pods). Our quantitative and qualitative user data correspond with literature on retail sales data and online search engine trends that followed the flavor bans. Future research is needed in order to evaluate black market/bootleg sales and the potential impacts on users and public health.

## Figures and Tables

**Table 1:** Within-subject changes in flavor usage after FDA regulation from data collected at survey time-point 3 (S3) (April 2020).

Flavor	S3 Previous Flavor Used (*n* = 86)	S3 Current Flavor Used (*n* = 86)	*p*-Value
Virginia Tobacco, % yes (*n*)	14.0 (12)	17.4 (15)	<0.01
Classic Tobacco, % yes (*n*)	4.7 (4)	10.5 (9)	<0.01
Menthol, % yes (*n*)	11.6 (10)	37.2 (32)	<0.01
Mint, % yes (*n*)	26.7 (23)	16.3 (14)	<0.01
Mango, % yes (*n*)	29.1 (25)	11.6 (10)	<0.01
Fruit, % yes (*n*)	8.1 (7)	4.7 (4)	<0.01
Crème, % yes (*n*)	3.5 (3)	1.2 (1)	1.0
Cucumber, % yes (*n*)	2.3 (2)	1.2 (1)	1.0

**Table 2:** Participant behaviors by survey time-point.

Participant Behaviors	Time-Point 1 July 2019 (*n* = 76)	Time-Point 2 January 2020 (*n* = 128)	Time-Point 3 April 2020 (*n* = 86)	*p*-Value
Current location to purchase JUUL pods is online, % yes (*n*)	23.7 (18)	20.3 (26)	15.1 (13)	0.38
Ever refilled pods, % yes (*n*)	19.7 (15)	34.4 (44)	11.6 (10)	<0.01
Ever experienced issues with purchasing genuine pods, % yes (*n*)	14.5 (11)	18.8 (24)	18.6 (16)	0.71
Ever intentionally purchased generic pods, % yes (*n*)	7.9 (6)	17.2 (22)	11.6 (10)	0.12
Reason purchased generic pods because of more flavor options, % yes (*n*)	50.0 (3/6)	40.9 (9/22)	70.0 (7/10)	0.39

**Table 3:** Joint Display of Quantitative and Qualitative Findings.

Overall Finding (Impact of FDA Ban)	Quantitative Finding	Qualitative Finding
Users were not impacted	Prior to any flavor restrictions, almost a quarter (23.7%) of participants already used tobacco or menthol products.	Theme: Users were not affected because they already used tobacco or menthol flavors. “I know some people really get hung up on those flavors. It’s never been a huge draw for me” “I’ve always only preferred the menthol and that’s the only one that I’ve used”
Users switched to available products	There was a significant reduction in mint use and a significant increase in menthol after the FDA ban.	Theme: Users easily transitioned from mint to menthol because both contained nicotine. “I did really like the mint ones. That was my go to, but the menthols are just as good” “You just adapt… so you get nicotine however you can get it”
Users started using products available in their preferred flavor	After the FDA ban, more than 10% of participants reported using generic products.	Theme: Users sought generic pods (or disposable products) when their preferred flavors were not available. “I always a hundred percent of the time, if it’s available, choose a generic that has a better flavor over the actual JUUL pod” “When I feel like I want to reward myself, I tell myself that I’ll get like a flavored Puff Bar.”
Users continued use of banned flavors	After the FDA ban, about 35% of participants reported continuing to use banned flavors.	Theme: Participants stock-piled banned flavors or found locations that continued to sell the products. “So it made me kind of hoard them.” “There were like a few stores I had around here that were like sketchy and like secretly had them.”

## Data Availability

The data presented in this study are available on request from the corresponding author. The data are not publicly available due to participant consent.

## Supplementary Materials

The following are available online at https://www.mdpi.com/1660-4601/18/1/207/s1, Table S1: Participant Characteristics.
