# Supplementary material for: Response to Flavored Cartridge/Pod-Based Product Ban among Adult JUUL Users: “You Get Nicotine However You Can Get It”
Source: Int J Environ Res Public Health. 2020 Dec 30;18(1):207. doi: 10.3390/ijerph18010207 (PMC7795757; doi:10.3390/ijerph18010207)
Supplement: Supplementary file 1 [file ijerph-18-00207-s001.pdf]

Supplemental Table 1: Participant Characteristics

	Quantitative Sample (n=290)	Qualitative Sample (n=16)
Age (SD) (median) (range)	31.7 (8.2) (30) (18-67)	33.8 (10.1) ( ) (23-59)
% young adult (aged 18-25) (n)	21.7 (63)	18.8 (3)
% male (n)	63.7 (185)	56.3 (9)
% current smoker (n) (timeframe)	18.3 (53) (in the past 30 days)	56.3 (9) (in the past 90 days)
% current JUUL user in the past 30 days	100.0 (290)	93.8 (15)
Mean days used JUUL in the past 30 (SD)	24.0 (8.3)	-
Mean JUUL times used per day (SD)	8.7 (9.2)	-
